# Geographic and socioeconomic inequities in cesarean delivery rates at the district level in Madhya Pradesh, India: A secondary analysis of the national family health survey-5

**DOI:** 10.1080/16549716.2023.2203544

**Published:** 2023-05-04

**Authors:** Rohini Dutta, Shagun Tuli, Minal Shukla, Priti Patil, Alex J van Duinen, Neil Thivalapill, Rakhi Ghoshal, Anusha Jayaram, Nobhojit Roy, Anita Gadgil

**Affiliations:** a Program in Global Surgery and Social Change, Department of Global Health and Social Medicine, Harvard Medical School, Boston, MA, USA; b Mary Horigan Connors Centre for Women’s Health and Gender Biology, Brigham and Women’s Hospital, Boston, MA, USA; c Department of General Practice, School of Medicine, University of Limerick, Limerick, Ireland; d Department of Obstetrics and Gynecology, Public Health Consultant, India; e Department of Statistics, Bhabha Atomic Research Centre Hospital, Mumbai, India; f Department of Public Health and Nursing, Norwegian University of Science and Technology, Trondheim, Norway; g Department of Surgery, St. Olavs Hospital Universitetssykehuset Trondheim, Trondheim, Norway; h Northwestern University Feinberg School of Medicine, Chicago, IL, USA; i Gender Equality Centre, CARE India, New Delhi, India; j Department of Surgery, Beth Israel Deaconess Medical Centre, Boston, MA, USA; k Department of Global Public Health, Karolinska Institute, Stockholm, Sweden; l The George Institute for Global Health, New Delhi, India; m Department of Surgery, Bhabha Atomic Research Centre Hospital, Mumbai, India

**Keywords:** Cesarean delivery rates, wealth quintiles, health disparity, global surgery, Global women's health

## Abstract

**Background:**

In India, caesarean delivery (CD) accounts for 17% of the births, of which 41% occur in private facilities. However, areas to CD in rural areas are limited, particularly for the poor populations. Little information is available on state-wise district-level CD rates by geography and the population wealth quintiles, especially in Madhya Pradesh (MP), the fifth most populous and third poorest state.

**Objective:**

Investigate geographic and socioeconomic inequities of CD across the 51 districts in MP and compare the contribution of public and private healthcare facilities to the overall state CD rate.

**Methods:**

This cross-sectional study utilised the summary fact sheets of the National Family Health Survey (NFHS)-5 performed from January 2019 to April 2021. Women aged 15 to 49 years, with live births two years preceding the survey were included. District-level CD rates in MP were used to determine the inequalities in accessing CD in the poorer and poorest wealth quintiles. CD rates were stratified as <10%, 10–20% and >20% to measure equity of access. A linear regression model was used to examine the correlation between the fractions of the population in the two bottom wealth quintiles and CD rates.

**Results:**

Eighteen districts had a CD rate below 10%, 32 districts were within the 10%–20% threshold and four had a rate of 20% or higher. Districts with a higher proportion of poorer population and were at a distance from the capital city Bhopal were associated with lower CD rates. However, this decline was steeper for private healthcare facilities (R2 = 0.382) revealing a possible dependency of the poor populations on public healthcare facilities (R2 = 0.009) for accessing CD.

**Conclusion:**

Although CD rates have increased across MP, inequities within districts and wealth quintiles exist, warranting closer attention to the outreach of government policies and the need to incentivise CDs where underuse is significant.

## Introduction

Caesarean delivery (CD) is a life-saving obstetric surgical procedure, and universal access to safe CD is a prerequisite for improving global maternal and foetal outcomes [1]. While the ideal population-based CD rates between 10–19% are considered sufficient to fulfill the requirements of the population, large regional variations in CD rates disproportionately affect low- and middle-income countries (LMICs) [2,3]. Caesarean rates exceeding 20% are seen in high-income countries (HICs) such as the United States and the United Kingdom [4]. In contrast, South-East Asian countries, such as Nepal, have low CD rates (5%), comparable to consistently low rates across the African continent, averaging between 4% and 6% in 2015 [5,6]. Furthermore, CD rates are differentially affected by factors, such as socioeconomic development, literacy, urbanisation, and the availability of skilled personnel in a healthcare facility [2]. Evidence suggests that more educated women, residing in urban areas are more prone to use private facilities for CD, compared to their less educated and financially disadvantaged counterparts in rural regions [3,6,7].

In India, CD accounts for 17% of the births, of which 41% occur in private facilities. A steady increase in the nationwide CD rate has been observed over the last few decades, which is primarily attributed to fee-for-service private healthcare facilities [6,7]. This increment varies within the Indian states and is not accompanied by a proportionate and concomitant decrease in maternal and neonatal mortality [8,9]. The highest maternal mortality ratio (MMR) is found within the rural areas of poorer states (397 per 100,000 live births), where direct obstetric causes account for the majority of maternal deaths [9]. Further, India as a nation shows varying trends of CD rates across states. For example, the CD rate in the state of Tamil Nadu (southern India, an affluent state) was almost two times higher (35.8%) than the nation’s average (19.2%). By comparison, in Bihar (Eastern India, a relatively poorer state) it was three times lower (7.4%) than the national average [10].

Madhya Pradesh (MP) located in central India, is designated as an Empowered Action Group state (EAG) owing to its poor socioeconomic development status [11]. It is also the fifth-most populous state of India [12]. With 173 per 100,000 live births, the state MMR was the third highest in the country (2018–2020) [13]. As per the WHO thresholds, its current state-wide CD rate of 12.1% is indicative of meeting the needs of obstetric patients [2,14]. However, with 72% of the population being predominantly rural, disaggregation of the data is needed to investigate the high MMR due to obstetric causes despite adequate CD rates [15].

Aggregating data from multiple districts to determine the prevalence of non-medically indicated CD may hide the differences and variations that exist between districts. For example, in another EAG state, Bihar, disaggregation of the statewide CD rate revealed potential overuse of CDs in districts with less poorer populations [15]. This highlights the important systemic inequalities that are unjust and avoidable discrepancies in CD rates within a population [16]. Measuring inequalities in CD use can help identify high-risk population subgroups and allow governments to form policies that can reduce inequities. Therefore, this study aims to assess the change in CD rates over time from 2015–16 to 2019–21 in MP using the national family health survey data. Secondarily, it investigates geographic and socioeconomic inequities of CD among MP’s 51 districts. Finally, it compares the contribution of public and private healthcare facilities to the CD rates in the state of MP and demonstrates the variation in inequitable access to CD between 2015–2021.

## Methods

### Study setting

MP is in central India. With a population of 72 million people (48.2% women, sex ratio of 970), 72.4% reside in rural areas and 30% of the state population is illiterate [12]. At 163, the MMR of MP is among the highest in India [13]. The state is divided into 51 administrative districts, which are further classified into 7 divisions viz. Bhopal, Indore, Gwalior, Rewa, Ujjain, Jabalpur and Sagar.

**Figure 1.**
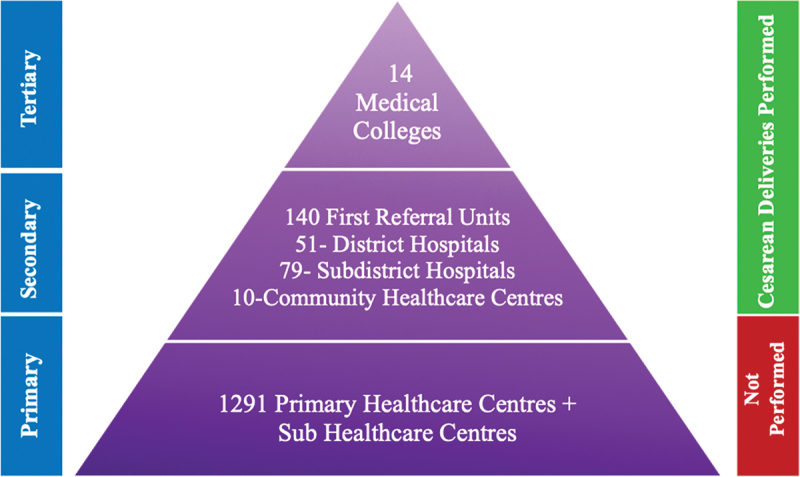
Madhya Pradesh government’s three-tier healthcare system [17].

Of the total districts, 13 have government medical colleges (Bhopal, the state capital, has 2 medical colleges) and one medical college at each divisional headquarter providing tertiary-level healthcare facilities complementing the first referral units (FRUs) in providing comprehensive obstetric emergency care. The government healthcare system in MP consists of primary, secondary, and tertiary care levels as depicted in Figure 1. Caesarean delivery is accessible at all levels except the primary healthcare centres, which lack surgical capacity.

### Data source

The National Family Health Survey (NFHS) is conducted over multiple rounds in a representative sample of households across India [14]. Data is collected through computer assisted personal interviewing [14]. The fourth and fifth survey rounds were conducted by the International Institute for Population Sciences, Mumbai in 2015–2016 and 2019–21, respectively, as designated by the Ministry of Health and Family Welfare (MoHFW), Government of India. The survey was conducted by the Development and Research Services Pvt. Ltd. (DRS) and the Indian Institute of Development Management [14].

The NFHS provides data on district-level institutional birth and CD rates in private and government facilities in MP. Additionally, antenatal care, delivery care, family planning services, and literacy rates among women are also included. For MP, the NFHS-5 data collection was done across 731,894 households, including 628,801 women aged 15–49. Specifically for Madhya Pradesh, 43552 households, and 48,410 women aged 15–49 were interviewed [14]. We considered the districts of MP as the unit of analysis for our study. Table 1 provides a comparison of key indicators on CD in MP to the national statistics.

**Table 1.** Caesarean delivery data in India vs. Madhya Pradesh.

NFHS-5 (2019–2021)	India	Madhya Pradesh
Institutional births (%)	88.6	90.7
Institutional births in public facility (%)	61.9	80.2
Births by cesarean delivery (%)	21.5	12.1
Births in a private health facility that were by cesarean delivery (%)	47.4	52.3
Births in a public health facility that were by cesarean delivery (%)	14.3	8.2

The NFHS-5 may be subject to potential biases, such as sampling, non-response, social desirability, and recall biases. To minimise this, NFHS-5 employed measures such as using a representative sample, maximising response rates, using anonymous questionnaires for sensitive questions, and verifying reported events with official records. Standardised questionnaires and procedures were also used to enhance the validity and reliability of the survey results.

### Variables

In this study, we have considered the district-wise CD rates as the output variable. The proportion of the population living in various districts of MP for poorest and poorer wealth quintiles mentioned in the state report of the NFHS was considered as the explanatory variable to find the inequality of the CD rates between the different districts. The wealth quintile is the measure of the economic status of the household which is constructed using household asset data via principle component analysis. The resulting wealth quintiles divide the population into five equal groups, with each group representing 20% of the population, based on their level of wealth [18]. The distribution of the proportion of populations within the poorest and poorer wealth quintiles was summarised for each district.

### Data analysis

The CD rates were stratified as <10%, 10–20% and >20% to compare with the threshold for ‘optimal’ caesarean delivery rate as per the WHO [2]. Less than 10% were considered inadequate, and >20% were considered excessive. The top five districts having population subgroups in the two richest wealth quintiles are Indore, Bhopal, Gwalior, Ujjain and Neemuch. The poorest districts are Dindori, Jhabua and Alirajpur [14]. The CD rates of NFHS-4 and NFHS-5 were graphically compared using maps to depict the trends of each district. We aggregated the shares of population subgroups in the two poorest quintiles to compare districts. The relationship between CD rates and the proportion of the population belonging to the poorer and poorest wealth quintile group was evaluated using the linear trend lines created in Microsoft Excel. Maps were created using the QGIS V.3.12, Open Source Geospatial Foundation Project.

## Results

MP observed an overall increase in the institutional birth rate of 10% and an increase in the CD rate of 3.5% (from 8.6% in 2015–16 to 12.1% in 2019–21). More than half of all births in private healthcare facilities were CDs (52.3%), whereas only 8.2% of all deliveries in the public sector were CDs.

### District-level disparities in cesarean delivery rates across MP

**Figure 2.**
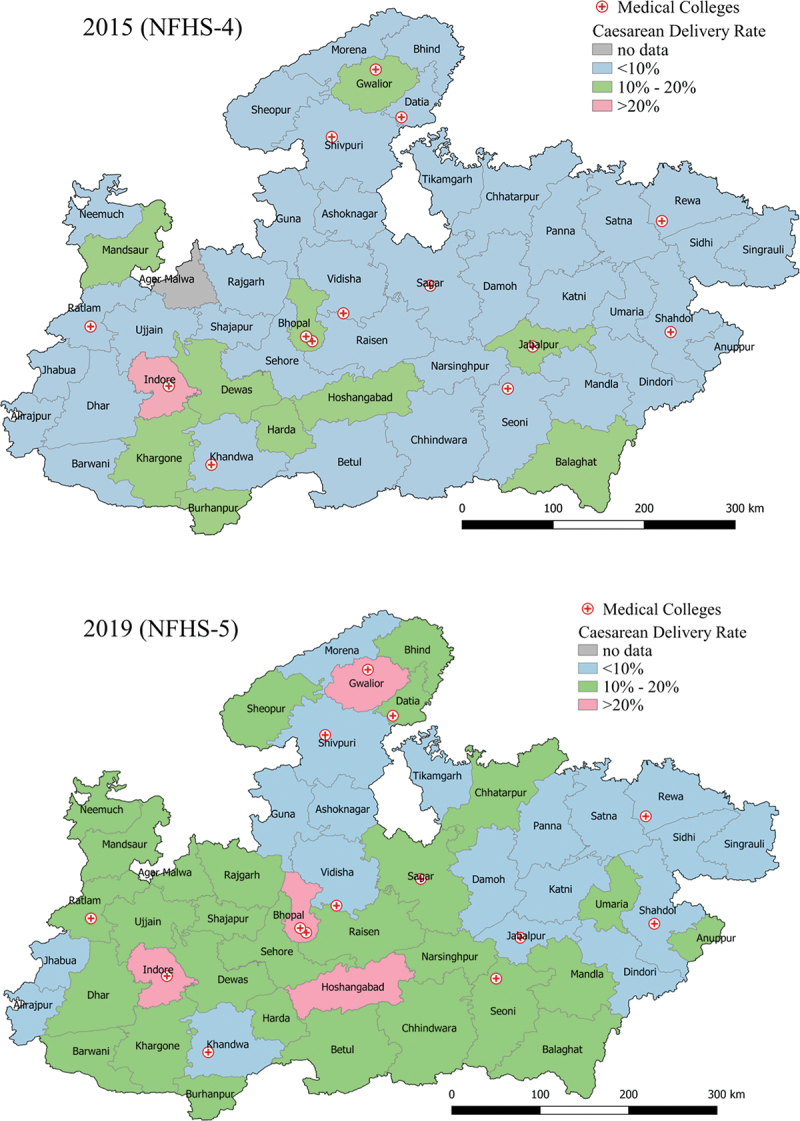
Geographical depiction of cesarean delivery rates in the districts of MP in 2015 (2a (2015–16)) and 2019 (2b (2018–19)).

There was a decline in the number of districts with CD rates below 10% from 38 in 2015 to 19 in 2019. When considering the 10% threshold for an ‘optimal’ CD rate for a population [2], six districts (Betul, Sheopur, Umaria, Barwani, Shajapur and Mandla) had a CD rate of 10%, 18 districts had a rate below 10% while 26 districts were above the 10% threshold. Four districts (Gwalior, Bhopal, Indore and Hoshangabad) had a CD of 20% or higher (Figure 2)

Disaggregation at the district level shows us an unequal increase in the state CD rates from 2015–16 to 2019–21. When compared with the state, an average increase of 12.1% is seen. Twenty-five out of the 51 districts showed a greater increase in CD rates, ranging between 18.3% and 22.5%. The Jhabua district had an equal rate as the state, whereas 17 districts were below the state average. Five districts (Jabalpur, Katni, Khandwa, Tikamgarh and Dewas) showed a decrease in CD rates indicating that the overall state average increase is not substantial in ascertaining service provision (Figure 2).

Forty-five districts had an overall rise in CD rates, of which 18 districts had an increment of ≥5% (range: 5.2%–10.7%). On the contrary, five districts experienced a reduction in CD rates by 1.3–11.5%. Although two out of the five districts (Khandwa and Jabalpur) have medical colleges and access to tertiary health care services, Jabalpur has witnessed the steepest decline in the CD rate from 18.7% in 2015 to 7.2% in 2019. The remaining 13 districts with medical colleges have increased CD rates of which Hoshangabad has the highest rise of 10.7%. Other districts with medical colleges established most recently show an incremental trend of CD rates; Chhindwara from 8.3%, Datia 6.0%, Ratlam 5.6%, Vidisha 4.8%, Shahdol 3.1% and Shivpuri 2.7% (2015–16 to 2019–21).

**Figure 3.**
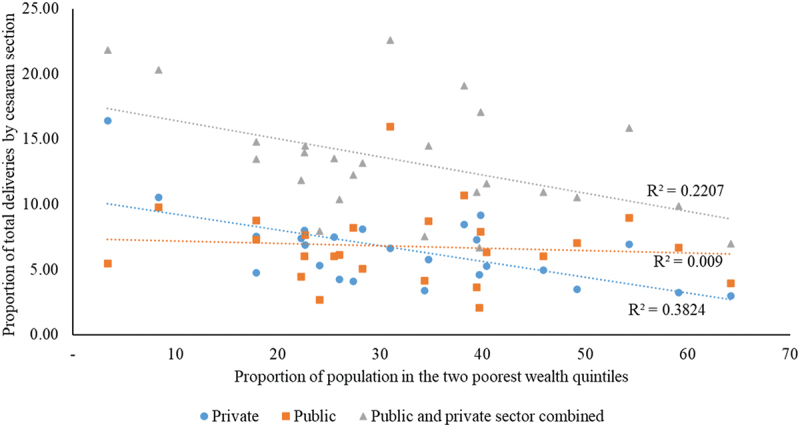
Cesarean delivery rates by district proportion of poorest and poorer wealth quintiles population in 51 districts of MP in 2019–21.

According to NFHS-5, 30–77% of the population in the poorest two wealth quintiles reside in 35 out of the 51 districts in MP, while 16 districts have less than 30% of the population in the poorest wealth quintile. CD rates in both private and public health care facilities decline as we move from districts with relatively rich to poorer populations (Figure 3) represented by R2 = 0.221, *p* = 0.018. However, this decline is steeper for private healthcare facilities (R2 = 0.382, *p* = 0.01) than for public healthcare facilities, which indicate almost no correlation (R2 = 0.009 *p* = 0.652) in accessing CD. Though showing a decline, almost no correlation (0.009) is noted for the public sector.

## Discussion

Though the CD rates have increased from 8.2% in 2015–16 to 12.1% in 2019–21 in MP, there remains a stark disparity in access to caesarean delivery. With regard to the primary aim, our findings reveal a variation across districts due to factors, such as wealth status and geographic constraints. The number of districts in MP with CD rates more than the WHO recommended range has increased to four districts with a rate of 20% or more in 2019–21 from one in 2015–16. Exploring our secondary objective shows us that districts without medical colleges and with poorer populations showed a decline in CD rates and this decline was steeper in private healthcare facilities, while CD rates across public healthcare facilities remained steady.

### Wealth status as a social determinant in accessing cesarean delivery

Disaggregation of the CD rates with respect to the wealth quintiles highlights existing inequities within the public and private health systems. As of 2019, the estimated number of private and public healthcare facilities in MP was 506 and 465, respectively. However, the strong negative correlation suggests that access to private facilities for CDs may be a challenge for the poorer populations [19]. Though showing a decline, a much weaker correlation is noted for the public sector, highlighting that these services remain steadfast across districts with increasing proportions of poorer populations.

Globally, similar trends have been observed in other LMICs [20,21]. Studies in Tanzania and Ethiopia have concluded that the economic status of women is a key determinant of accessing CDs [20]. In these settings, financial constraints have been identified as a major factor hindering access to CD [20]. The Government of India has introduced schemes such as Janani Suraksha Yojana (JSY), and Ayushman Bharat that provide free access to CDs in public as well as accredited private setups to the poor [22,23]. These nationally funded schemes aim to decrease neonatal and maternal mortality rates in the country, especially in low-performing states [22–24]. In addition to the provision of free CDs, there are cash incentives for the poor when availing CDs from public health facilities. Further, the uptake of government schemes lags behind across private health facilities due to a lack of awareness about these among the population. This leads to higher out-of-pocket expenditure for CDs in private facilities, making the procedure inaccessible to socioeconomically disadvantaged populations. The increasing CD rates reflect the utilisation of private facilities by the populations in the higher wealth quintile, leaving out the poorer populations [24].

The granular reasons behind continued lower CD rates in public health facilities and increasing rates in private facilities require further exploration. Our results indicate that CDs continue to be a privilege for the wealthier populations despite an overall increase or a simultaneous existence of overuse and underuse of the procedure, as is also noted in the data disaggregation of our study. A study from Peru found CD rates to be higher in private facilities as compared to public [25]. However, the individual factors leading to the outcome of a CD were unique; a higher wealth quintile was associated with CDs in public set-ups [25].

### Geographic inequities in access to CD

Madhya Pradesh presents a highly diverse geographical landscape. There are 20 tribal, and 23 high-priority districts^1^^1^Government of India has identified high priority districts (HPD) in each state to ensure equitable health care and to bring about sharper improvements in health outcomes. The bottom 25% of the districts in every state according to the ranking of districts based on composite health index have been identified as HPDs [31]. with varied terrains owing to several hill ranges and massive riverbeds [26]. Furthermore, the population in MP is largely rural (72.37%) with many tribal communities in the state [27]. An analysis of the NFHS-4 (2015–16) showed that 61.1% of the women in MP, used the JSY scheme out of which 38.5% were scheduled caste (SC) and scheduled tribe (ST) and a majority (61.1%) were non-SC/ST [28]. Studies from Bihar and Tamil Nadu also revealed caste-based trends in access to CDs in public healthcare facilities [10]. Though the Government of India has been focusing on improving services and access for tribal areas, the unmet need is high. A common barrier for tribal women to access CDs is the dearth of skilled obstetricians and anaesthesiologists and comprehensive emergency obstetric and neonatal care (CEmONC) [29]. A rudimentary analysis to understand how these inequities intersect with wealth as a social determinant is warranted.

Further, MP is landlocked and has limited access to paved roads in certain areas which may have contributed to the inequities we observe [30]. Our study highlighted clusters of districts in the East and North of MP with lower CD rates than the rest of the district (Figure 2). A potential reason for the less-than-optimal CD rates could be that these districts fall under the Northern Hill region and the Satpura Hill range [27]. MP shares its borders with five states: Uttar Pradesh in the north, Rajasthan in the west, Maharashtra in the south, Gujarat in the east and southwest and Chhattisgarh in the east. We observed similarities in CD rates in the neighbouring smaller districts of MP with the respective states in NFHS-5. These districts had lower CD rates as compared to the overall states such as Rajasthan 10.4% (districts of Jhalawar 8.6%), Gujarat 21% (district of Dahod 6.1%) & Maharashtra 25.4% (district of Nandurbar 7.9%). These observations further highlight that the geographic inequity we observe in MP potentially traverses state borders. Hence, national policy reforms must take into consideration India’s diverse geographic topography and the challenges they pose in accessing CDs.

### Research and policy implications

This study highlights the district-wise CD inequities that exist in a state with 23 high-priority districts [31]. Government efforts have been geared towards providing access to this procedure through policies, such as JSY, JSSK for more than a decade (2005, 2011), yet national policies, such as Ayushman Bharat that are not specific to maternal health are fairly new (launched in 2018) [22–24]. The contribution of these different policies on CD rates and outcomes may require a national cross-policy analysis with a focus on low-performing states and high-priority districts.

A similar study in Bihar state also observed socioeconomic disparities in CD rates, suggesting that such analysis is warranted at a national level [15]. Future work should focus on assessing the association of state-wise CD rates with maternal outcomes and looking at a granular level, the factors affecting CD rates at the regional, state and district level. This will require continuous surveillance and monitoring of CD rates using the Robson classification.

### Strengths and limitations

The study utilises secondary data obtained from the NFHS, the largest and most dependable survey data source at the household level in India. Additionally, the study offers a more detailed perspective on the distribution of CDs in MP at the district level and its correlation with economic status. However, in India, due to a lack of comprehensive civil registration and hospital data, the proportion of institutional CDs and other publicly available data from the NFHS was used to conduct a secondary analysis. Although the NFHS has been beneficial in providing an initial comprehension of CD rate trends, the fact that data is collected every three to five years is a drawback of this study, as it prevents real-time evaluation. Other limitations include that the regression model looks at district-level data with regard to wealth quintiles, hence individual factors contributing to inequities may have been overlooked or misinterpreted in the analysis. Furthermore, there is no consensus on the exact CD rate that is known to have the greatest benefit in lowering maternal mortality while posing the least risk of avoidable operations. As a result, the decision to establish cut-offs at 10% and 20% were made pragmatically, taking into consideration the available evidence. In NFHS-5, CD rates in private facilities in 25 districts (Alirajpur, Anuppur, Ashok Nager, Balaghat, Barwani, Betul, Bhopal, Damoh, Dhar, Dindori, Jabalpur, Katni, Khandwa, Khargone, Mandla, Mandsaur, Raisen, Rewa, Satna, Shahdol, Shivpuri, Sidhi, Singrauli, Tikamgarh and Umaria) were unavailable. Additionally, district-level data for maternal mortality rates were unavailable due to which we were not able to assess the correlation of CD rates with MMR.

## Conclusion

The study highlights the existing inequities by disaggregating data at the district level. Though an overall increase has been observed in Madhya Pradesh from 8.2% in 2015–16 to 12.1% in 2019–21, this increase has not been uniform across the state. A nuanced understanding of barriers that speak to the on-ground challenges, socioeconomic, geographic, cultural, political, or others is warranted to develop policy reform. Adopting a granular approach to understanding CD trends should be mainstreamed to inform schemes that incentivise CDs.

Increasing national averages while retaining equity in countries with exceptionally low rates is a significant, yet challenging task. As a first step, it is essential to have continuous and thorough monitoring of CD rates at the national, regional, sub-regional, and institutional levels, specifically focussing on which women are undergoing CDs and which are not [32]. Future work should look at collecting primary institutional-level data on the indications and outcomes of CDs in each state.

## Data Availability

The data is available in a public, open-access repository, and the corresponding author can also share it. We investigate the geographic and socioeconomic inequities of CD across the 51 districts in MP and compare the contribution of public and private healthcare facilities to the overall state CD rate.

## Paper context

Caesarean delivery accounts for 17% of the births in India, of which 41% occur in private facilities. District-wise disaggregation of these rates against geographic and socio-economic determinants reveals inequities in access to this procedure in the state of Madhya Pradesh where overall caesarean rates have increased from 8.2% to 12.1% from 2015 to 2021. Understanding the barriers at the grassroot level can help guide policy to encourage equity in government programmes that incentivise access to cesarean deliveries.
